# Increased Voluntary Activation of the Elbow Flexors Following a Single Session of Spinal Manipulation in a Subclinical Neck Pain Population

**DOI:** 10.3390/brainsci9060136

**Published:** 2019-06-12

**Authors:** Mat Kingett, Kelly Holt, Imran Khan Niazi, Rasmus Wiberg Nedergaard, Michael Lee, Heidi Haavik

**Affiliations:** 1 Centre for Chiropractic Research, New Zealand College of Chiropractic, Auckland 1060, New Zealand; Mat.Kingett@nzchiro.co.nz (M.K.); kelly.holt@nzchiro.co.nz (K.H.); imran.niazi@nzchiro.co.nz (I.K.N.); r.nedergaard@rn.dk (R.W.N.); 2 Faculty of Health & Environmental Sciences, Health & Rehabilitation Research Institute, AUT University, Auckland 0627, New Zealand; 3 Centre for Sensory-Motor Interactions (SMI), Department of Health Science and Technology, Aalborg University, 9220 Aalborg, Denmark; 4 Mech-Sense, Department of Gastroenterology and Hepatology, Aalborg University Hospital, 9000 Aalborg, Denmark; 5 Graduate School of Health, Discipline of Physiotherapy, University of Technology Sydney, Sydney, NSW 2007, Australia; Michael.Lee-2@uts.edu.au

**Keywords:** spinal manipulation, voluntary activation, maximum contraction force, elbow flexors, interpolated twitch technique (ITT)

## Abstract

To investigate the effects of a single session of spinal manipulation (SM) on voluntary activation of the elbow flexors in participants with subclinical neck pain using an interpolated twitch technique with transcranial magnetic stimulation (TMS), eighteen volunteers with subclinical neck pain participated in this randomized crossover trial. TMS was delivered during elbow flexion contractions at 50%, 75% and 100% of maximum voluntary contraction (MVC) before and after SM or control intervention. The amplitude of the superimposed twitches evoked during voluntary contractions was recorded and voluntary activation was calculated using a regression analysis. Dependent variables were analyzed with two-way (intervention × time) repeated measures ANOVAs. Significant intervention effects for SM compared to passive movement control were observed for elbow flexion MVC (*p* = 0.04), the amplitude of superimposed twitch (*p* = 0.04), and voluntary activation of elbow flexors (*p* =0.03). Significant within-group post-intervention changes were observed for the superimposed twitch (mean group decrease of 20.9%, *p* < 0.01) and voluntary activation (mean group increase of 3.0%, *p* < 0.01) following SM. No other significant within-group changes were observed. Voluntary activation of the elbow flexors increased immediately after one session of spinal manipulation in participants with subclinical neck pain. A decrease in the amplitude of superimposed twitch during elbow flexion MVC following spinal manipulation suggests a facilitation of motor cortical output.

## 1. Introduction

Several research studies have reported transient increases in voluntary strength in various muscle groups following spinal manipulation [1,2,3,4,5,6,7,8,9,10]. This has been demonstrated in healthy participants [1,4,5,8] as well as those with subclinical neck pain [7], knee pain [9], chronic neck pain [10], and low back pain [11]. However, the precise mechanisms mediating the improvement in strength following spinal manipulation remain elusive.

Previous research has also demonstrated that spinal manipulation induces neuroplastic changes that alter somatosensory processing, sensorimotor integration and motor control of the body [12]. As such, it is plausible that similar neurophysiological mechanisms may also contribute to strength improvement following spinal manipulation [13].

In order to assess the potential neurophysiological mechanisms associated with spinal manipulation without the confounding effect of pain, several research studies have been conducted in participants with subclinical neck pain [14,15]. Subclinical neck pain participants have reoccurring neck dysfunction, such as mild neck pain, stiffness or ache, with or without a previous episode of neck trauma [15]. However, patients in this subclinical pain group do not have constant symptoms and are not in pain at the time of investigation, thus providing a unique opportunity for scientists to explore the effects of various manual therapeutic interventions without the confounding factors of changes in pain levels. Pain alone has been shown to impair centrally mediated mechanisms of neural drive [16]; therefore, in order to provide novel insights into the neurophysiological effects of spinal manipulation, studies must be performed in pain-free participants. As such, asymptomatic patients with subclinical neck pain are ideal for this purpose. Previous studies in participants with subclinical neck pain have demonstrated reduced cervical range of motion [17], reduced cervicocephalic kinesthetic sensibility [18], altered upper limb proprioception [15], alterations in cortical and cerebellar processing [19,20], and impaired ability to perform cognitive tasks [14]. Furthermore, several studies have demonstrated that spinal manipulation can improve upper limb proprioception [15], increased cerebellar-M1 communication [19,20], increased lower limb strength [7] and prevent development of fatigue during repeated maximal contractions [7].

Although it has been hypothesized that the improvement in muscle strength following spinal manipulation may be caused by an increase in central neural drive [3,4,9,10,11,21], the precise underlying mechanism and the site of neural interactions remain to be elucidated. The “interpolated twitch” technique (also known as “twitch interpolation”) is a reliable method of quantifying neural drive [22,23]. This technique is commonly used to assess the completeness of skeletal muscle activation during voluntary contractions [23]. It involves a supramaximal electrical stimulation to the nerve trunk or intramuscular nerve branches during a maximum voluntary contraction. The supramaximal electrical stimulus activates all motor units synchronously, including those that are firing sub-maximally (that are not in a refractory period). Incomplete voluntary activation is evident from a ‘twitch-like’ increment in force during a maximal voluntary contraction (MVC) [23]. More recently, twitch interpolation with transcranial magnetic stimulation (TMS) was developed to assess motor cortical drive [24]. This technique has been shown to be reliable in the elbow flexors [24], wrist extensors [25], and knee extensors [26]. Similar to twitch interpolation using electrical stimulation, if a cortical stimulus could elicit extra force during a MVC, it would indicate that output from the motor cortex was sub-maximal [27] and suggests that the failure of neural drive must be occurring at or above the level of the motor cortex [24,28]. Twitch interpolation using electrical stimulation has previously been used to investigate changes in neural drive to the quadriceps following sacroiliac joint manipulation in participants with anterior knee pain [9]; however, no study to date has used twitch interpolation with TMS to investigate the effects of spinal manipulation in participants with subclinical neck pain.

The aim of the current study was to investigate the effects of a single session of spinal manipulation on voluntary activation and strength of the elbow flexor muscles using sensitive twitch interpolation techniques with TMS and brachial plexus stimulation.

## 2. Methods

### 2.1. Subjects

Eighteen participants with subclinical neck pain (13 males) aged between 18 and 40 (Mean 24.9 ± 4.1) participated in this study. The participants gave written informed consent, which conformed to the Declaration of Helsinki, and the study was approved by the Northern Y Regional Ethics Committee of New Zealand. Participants were included in the study if they had previously experienced neck pain, discomfort or neck ache that they had not sought treatment for. Participants were excluded from the study if they were currently in pain or had any pre-existing condition that prevented them from receiving spinal manipulation.

Each subject participated in two experimental sessions in which all the recordings (see details below in Section 2.2) were made pre and immediately post-intervention. Subjects either received spinal manipulation or a control (explained below in Section 2.4) in each session separated by at least one week. Our target population for this study was subclinical neck pain patients for several reasons: to avoid the confounding effect of current pain, to avoid any confounding effect from current or past treatment for more sever spinal problems and to ensure they were likely to actually need spinal manipulation (i.e., without a history of spinal problems there may not be any clinical reason to provide spinal manipulate to their spines). Maximal elbow flexion force and voluntary activation of the dominant elbow flexors (17 right-handed, 1 left-handed according to the Edinburgh handedness inventory [29]) were assessed before and after a passive movement control or spinal manipulation intervention session. The order of intervention (passive movement or spinal manipulation) was randomized.

### 2.2. Data Recording and Analysis

#### 2.2.1. EMG Recording

Standard surface electromyographic (EMG) techniques were used to record electrical muscle activity from the biceps brachii and triceps using bipolar surface electrodes (20 mm Blue Sensor Ag/AgCl, AMBU A/s, Denmark) with an inter-electrode distance of 1.5 cm. Surface EMG was amplified using a custom-built EMG amplifier and was recorded using a CED Power 1401 mk 2 data acquisition board at 5KHz.

#### 2.2.2. Electrical and Transcranial Magnetic Stimulation

The brachial plexus was stimulated with a single pulse electrical stimulus (pulse width 200 µs) via a constant current stimulator (Digitimer, model DS7AH, Hertfordshire, United Kingdom). The maximum compound muscle action potential (Mmax) from the biceps brachii was measured both at rest and during isometric contraction. The stimulus intensity was then set at 120% of that required to produce the maximal response.

A Magstim 200^2^ magnetic stimulator with a figure of 8 coils (outside diameter of each loop 70 m) was used to elicit motor evoked potentials (MEP) in the biceps brachii. The coil was held over the motor cortex at 45° to the sagittal plane in the optimal position to elicit MEP’s of the biceps brachii muscle. The position of the coil was then marked directly on the scalp and its relationship to anatomical landmarks noted. The resting motor threshold was determined to be the lowest stimulator intensity required to elicit a MEP lager than 50% of resting Mmax in at least three of five trials. EMG activity recorded over the bicep’s brachii was monitored during threshold trials and trials were excluded if EMG activity above background noise was detected.

The MEP and Mmax data were bandpass filtered (5 to 500 Hz) and sampled at 10 kHz with a power 1401 mk^2^ (CED, Cambridge, UK) interfaced with a computer running a custom-written signal program (CED, UK). The EMG data were amplified by a Grass 7P11 pre-amp with a gain of 1000. Peak to peak amplitudes of the waveforms were analyzed offline using a program that allowed the experimenter to set cursors at the beginning and end of each waveform. MVC amplitude was taken as the highest force reached pre-stimulation from any trial. The superimposed twitch (SIT) amplitude was measured by setting one cursor at the force immediately prior to stimulation and one at the highest force reached while the muscle was being stimulated.

#### 2.2.3. Force Recording and Measurement of Maximal Voluntary Contraction Force

Participants sat in a chair with their upper arm resting on a bench at a comfortable height with their elbow flexed to 90°. Their arm was then fixed into a custom-built apparatus by velcro straps over the length of the forearm. Forces produced were recorded via a calibrated load cell (LT1016 100 A & D Mercury, Thebarton, SA, Australia) set parallel to the bench. Signals were amplified using a Grass Direct Current preamplifier (Grass Instrument Co, Massachusetts, USA) with a gain of 1000 and low-pass filtered at 100 Hz. Output of the load cell was calibrated against known weights in Kg’s. For analysis, the force values were converted from volts to Kg’s. Particular attention was taken to make sure postural changes did not occur during the data collection sessions, as corticomotor excitability is known to change due to positional changes and orientation of the arm [30,31]. Participants were given time initially to familiarize themselves with the testing equipment and procedures and warm up with a series of sub-maximal elbow flexion contractions. After familiarization, participants were then asked to perform two brief (lasting 2–3 s) elbow flexion MVC’s. Real-time feedback of force was provided on a computer monitor (running a custom-written Spike 2 program) and standard verbal encouragements were given throughout to ensure maximal effort. A two-minute rest period between each MVC attempt was used to prevent fatigue. The participants were allowed to repeat any trial which they perceived to be sub-maximal. The larger of the two trials was taken as the baseline MVC.

##### Calculation of Voluntary Activation

Force traces were low-pass filtered at 100 Hz and recorded via a custom software program (Signal, Cambridge, UK). Analysis of raw recordings was performed offline. Initially, the peak force attained prior to the stimulus was measured and used to determine the MVC force. Subsequently, any additional change in force output produced by TMS was measured as the superimposed twitch (SIT). The resting twitch was estimated for each subject by extrapolating a linear regression between the SIT and voluntary forces at 100%, 75% and 50% of elbow flexion MVC. The y-intercept was then taken as the estimated resting twitch (ERT) [24,27]. This method provides a reliable measure of ERT and voluntary activation [25]. It is more reliable than using an experimental resting TMS twitch because both motor cortical and motor neuronal excitability vary with activity [32,33]. Voluntary activation was quantified by comparing the size of the SIT as a proportion of the ERT using the following formula [24,34]:

Voluntary activation (%) = (1 − SIT/ERT) × 100(1)

### 2.3. Experimental Protocol

**Figure 1:**
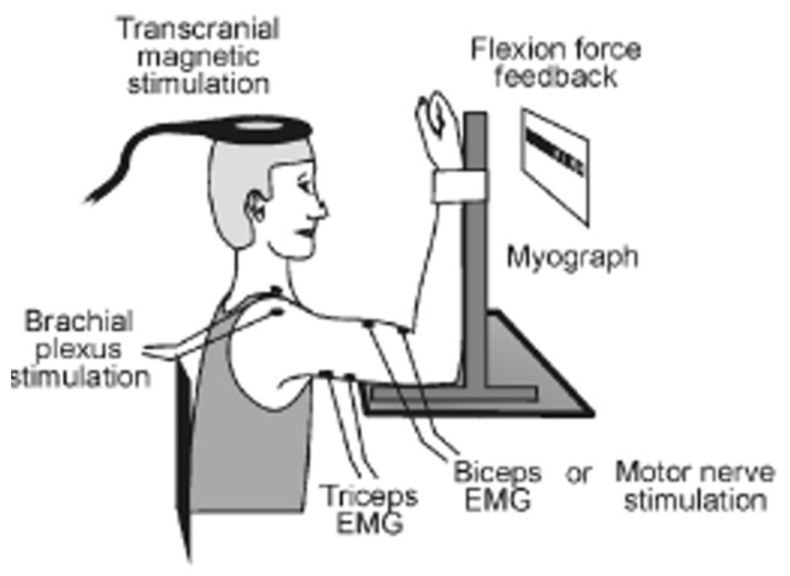
The experimental setup for this study was similar to a previous study (Todd et al., 2003, with permission); however, we used a figure of eight coils, not a circular coil for this current study.

The experimental protocol involved two sets of 4 voluntary isometric elbow flexion contractions (each lasting 2–3 s) which included two MVCs, one 75% MVC and one 50% MVC, in that order, with 4–5 s rest between each contraction. TMS was delivered over the contralateral motor cortex during the first MVC, 75% MVC and 50% MVC. A supramaximal electrical stimulus was delivered to the right Erb’s point during the second elbow flexion MVC, and the amplitude of the evoked twitch forces was recorded and analyzed offline (experimenter made sure that they were at a constant desired level before the stimulus was delivered). This protocol was repeated twice with a 2-minute rest. Real-time visual feedback of the voluntary force was displayed on a computer monitor to assist participants throughout the testing. Participants were asked to match the force as soon as prompted by a visual cue and hold that level of contraction until the magnetic or electrical stimulation was received, except in the case of the 100% MVC where participants were asked to surpass it (for setup, see Figure 1). During MVC’s, standardized verbal encouragement was provided to all participants to ensure maximal effort.

### 2.4. Interventions

#### 2.4.1. Spinal Manipulation

The experimental intervention consisted of spinal manipulation of dysfunctional spinal and sacroiliac joints (also known within the chiropractic profession as vertebral subluxations). Initially, the entire spine and pelvis were assessed for segmental dysfunction by a registered chiropractor with over 10 years of clinical experience. Clinical indicators used to assess for dysfunction were manually palpating for restricted inter-segmental motion, tenderness to palpation over relevant joints, any abnormal or blocked joint play, and palpable asymmetric inter-vertebral muscle tension. All of these characteristics are used by the chiropractic profession and other manual therapists as indicators of spinal dysfunction [35]. The spinal manipulations were all high velocity, low amplitude thrusts to the dysfunctional vertebral segments. Cervical manipulations were performed supine, thoracic manipulations were performed prone and lumbo-pelvic manipulations were performed in a side lying position or using a mechanically assisted drop table. The mechanical properties of this type of spinal manipulation have been investigated, and although the actual force applied to the spine depends on the clinician, the general shape of the force-time history is very consistent [36]. These spinal manipulation techniques has been previously employed by our research group to investigate the neurophysiologic effects of spinal manipulation [7,12,37].

#### 2.4.2. Control Intervention (Passive Movement)

The control intervention consisted of passive motion of the spine and moving the participant into a position that a chiropractor would normally use to deliver the spinal thrusts (i.e., a pre-manipulation position). This was performed by the same registered chiropractor that performed the manipulations but with care taken not to load any particular spinal joints to the end range as this is known to alter proprioceptive firing of the paraspinal tissues in anesthetized cats [38]. As the underlying mechanism(s) of spinal manipulation is thought to require the reaching of a certain mechanoreceptor ‘activation threshold’ to alter central motoneuronal or nociceptor excitability [39], the control experiment was performed with care to avoid providing a large mechanoreceptor input during the passive movement control. No manual thrust was applied during the control intervention. The control intervention was not intended to act as a sham manipulation, but to control for any physiological changes that cutaneous, muscular or vestibular input could impart during the same passive movements involved in preparing the participant for the spinal manipulative procedure. It also acted as a control for the time taken to carry out the spinal manipulation intervention and the stimulus necessary to collect the dependent measures of the study.

### 2.5. Statistical Analysis

Two-way repeated measures analysis of variance (ANOVA) was used to assess for differences between the effects of a single session of spinal manipulation or passive movement on elbow flexion MVC force, voluntary activation of the elbow flexors, and superimposed twitch amplitude during isometric elbow flexion with. Time (pre and post-intervention measures) and Intervention (spinal manipulation and passive movement control) were used as factors. Baseline group differences and post hoc pairwise comparisons were assessed using Tukey’s HSD tests, and an alpha level of 0.05 was used to determine statistical significance for all tests.

## 3. Results

### 3.1. Baseline Measurements

There were no significant differences between groups in baseline measurements of voluntary activation (VA), MVC force or superimposed twitch (See Table 1).

### 3.2. Superimposed Twitch

**Figure 2:**
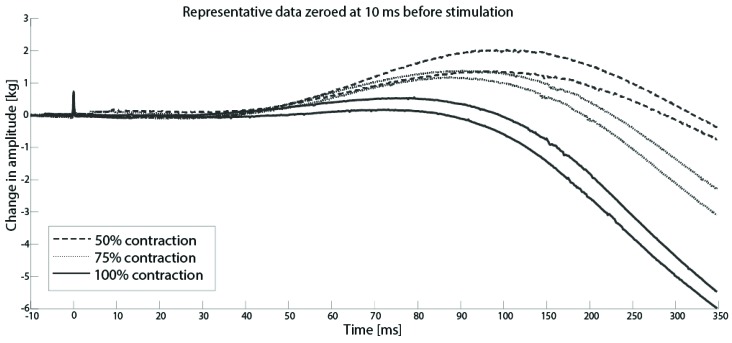
Data from a representative subject of force curves for single trial showing decreasing twitch force with increased voluntary contraction. (Force was zeroed at 10 ms before stimulus).

Figure 2 shows a representative subject performing voluntary elbow flexion contractions at 50%, 75% and 100% of MVC. As expected, the amplitude of the evoked twitches was greatest during the 50% MVC and smallest during the 100% MVC.

**Figure 3:**
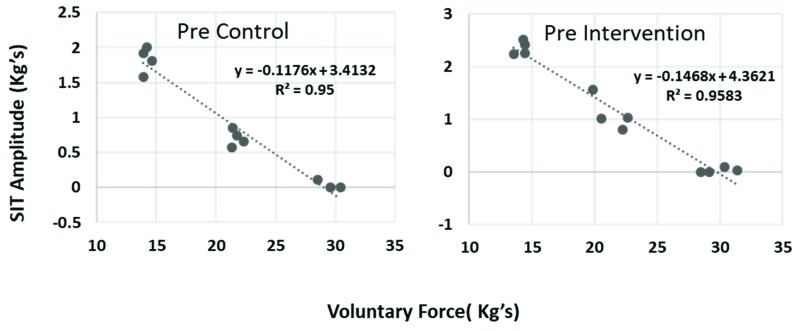
One representative subject’s data on two different days of linear regression analysis to calculate resting twitch.

For all participants, the size of elbow flexion SIT decreased linearly with increasing contraction force between 50% and 100% of MVC. Figure 3 shows raw data from a representative subject from one trial.

**Figure 4:**
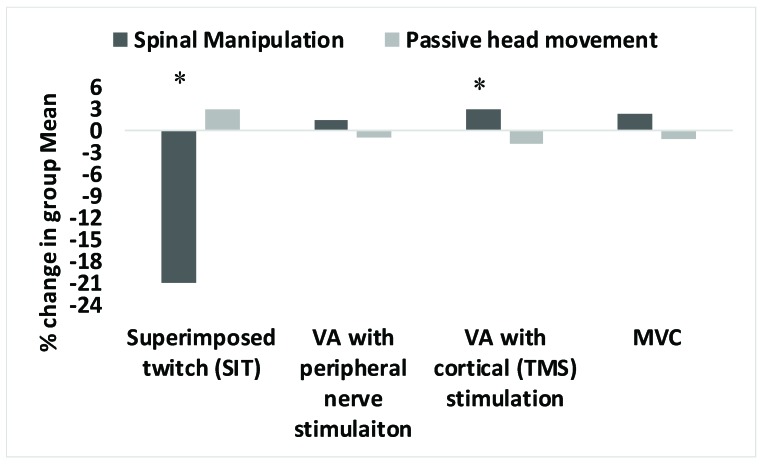
Percentage changes in group mean from pre to post of superimposed twitch (SIT), Voluntary activation (VA) with peripheral nerve (electrical) stimulation (*N* = 11) and cortical (TMS stimulation (*N* = 18) and MVC in the spinal manipulation (intervention) and passive movements (control) sessions across all participants. * represents *p* < 0.05.

There was a significant intervention by time interaction for the superimposed twitch at 100% MVC (F(1,17) = 4.50, *p* = 0.04, η*p*^2^ = 0.22). There was a significant mean group decrease of 20.9% in the superimposed twitch evoked during elbow flexion MVC following spinal manipulation (*p* = 0.005). Following the passive movement control there was a non-significant mean group increase of 2.9% in SIT (*p* = 0.79). (See Figure 4).

There were no significant between-group changes in the amplitude of SIT at 50% and 75% elbow flexion MVC following spinal manipulation and the passive movement control.

### 3.3. Voluntary Activation

There was a significant intervention by time interaction for voluntary activation of the elbow flexors following spinal manipulation (F(1,17) = 6.01, *p* = 0.03, η*p*^2^ = 0.26). Following spinal manipulation, there was a significant mean group increase of 3.0% (*p* = 0.0004) in voluntary activation of the elbow flexors measured via TMS, whereas following the passive movement control, there was a non-significant mean group decrease of 1.8% (*p* = 0.4). (See Figure 4). Out of 18, eleven participants were able to complete the electrical stimulation protocol because it was uncomfortable and non-tolerable. There was no significant intervention by time interaction for the superimposed twitch at 100% MVC (*p* = 0.5) with electrical stimulation.

### 3.4. MVC

There was a significant intervention by time interaction effect for spinal manipulation on elbow flexion MVC force (F(1,17) = 5.23, *p* = 0.04 η*p*^2^ = 0.24). The mean group MVC force changes during elbow flexion were not significant for either intervention but increased on average by 2.3% (*p* = 0.2) following spinal manipulation and decreased by 1.2% (*p* = 0.2) following the passive movement control. (See Figure 4).

## 4. Discussion

The primary aim of this study was to investigate the potential neurophysiological mechanisms underlying increases in strength following a single session of spinal manipulation in patients with subclinical neck pain. Using sensitive and reliable TMS twitch interpolation techniques, we have demonstrated that the size of the superimposed twitch evoked by TMS during elbow flexion MVC was decreased following spinal manipulation but not a passive movement control, and this resulted in an increase in voluntary activation of the elbow flexors. This is the first study to use TMS to assess changes in cortical voluntary activation following spinal manipulation in this population. In the current study, we found a small but significant increase in voluntary activation following spinal manipulation. The remainder of the paper will discuss the potential neurophysiological mechanisms underlying this improvement in voluntary drive and its clinical implications.

In the present study, the mean level of voluntary activation of the elbow flexors in asymptomatic patients with subclinical neck pain was approximately 90%, which is comparable to that reported previously in healthy volunteers by Todd et al., 2003 (93.6% ± 5.6). This confirms that the elbow flexors are relatively well-activated. The current study also demonstrated that voluntary activation of the elbow flexors was enhanced immediately following spinal manipulation directed to dysfunctional vertebral segments in participants with subclinical neck pain. In contrast, passive movements of the spine and re-positioning of the participants had no effect on voluntary activation. Our results suggest that spinal manipulation increased motor cortical drive to the elbow flexors. This finding supports previous research carried out in the soleus muscle where there was an increase in both MVC force and V wave amplitude following spinal manipulation, along with a significant decrease in both MVC force and V wave amplitude following the control intervention [7]. As V waves also reflect cortical drive, the current study is the second study to show increased cortical drive to a muscle following spinal manipulation in a sub-clinical neck pain population.

Niazi et al., 2015 sought to investigate whether spinal manipulation altered neural plastic changes involving cortical drive and the H-reflex pathway for the soleus muscle. They demonstrated that spinal manipulation resulted in increased MVC’s, with the increase measured using both surface electromyography and absolute force. They also found spinal manipulation increased the descending drive to the muscles, measured by an increased V wave amplitude, with the only changes to the H-reflex being a small significant decrease in the H reflex threshold. Following the control session with no manipulation there was a decrease in the EMG, force and voluntary drive with no changes in the H reflex pathway [7]. The authors therefore concluded that the improvements in EMG and force during MVC following spinal manipulation were likely due to an increase in descending drive and/or modulation in afferents other than those involved in the H-reflex [3,7]. The current study also found that spinal manipulation increased cortical drive to the elbow flexors.

Although it is difficult to explain the precise mechanisms mediating the increase in voluntary activation of the elbow flexors following the spinal manipulation, the site of neural interaction must have occurred at or above the level of the motor cortex, evident from the decrease in SIT during elbow flexion MVC [28]. Using TMS, one previous study has shown that spinal manipulation of dysfunctional joints can change central inhibitory and facilitatory drive to upper limb muscles in a muscle specific manner [40]. In this study, spinal manipulation of dysfunctional cervical joints increased short-interval intracortical facilitation (SICF) and decreased short-interval intracortical inhibition (SICI) in the abductor pollicis brevis muscle, along with a shortening of the cortical silent period (CSP). Interestingly, the same cervical manipulations lead to the opposite effects in the extensor indicis muscle, with a significant decrease in SICF, and a lengthening of the CSP. Although we have since shown that the CSP changes are not inhibitory in nature following spinal manipulation [41], the muscle-specific changes (in opposite directions) suggest that afferent input from the spine alters the net balance with which the CNS activates muscles. Our current finding with an increase in central drive despite no change the MVC supports this notion. It does suggest that the manipulations have led to greater central drive, and as this is not resulting in greater MVC, this suggests that the intrinsic muscle force itself is decreased after spinal manipulation.

Why might this be happening? It is possible that spinal manipulation ameliorated previous pain-induced maladaptive plastic changes to biceps motor control. Episodes of acute pain, such as after an injury, may initially induce plastic changes in the sensorimotor system [42]. Such plastic changes can become a “chronically progressive, functional, structural, and neurochemical/molecular make-over of the entire core of the somatosensory (and motor) brain” [42]. These changes do not only occur centrally and may also show up with a change in the intrinsic muscle activation pattern as well (that may be due to central and/or peripheral changes). For example, noxious stimulation of the upper trapezius muscle has been shown to result in a shift of the distribution of activity towards the caudal region of the muscle during performance of a repetitive lifting task [43]. This change in the distribution of activity to different regions of the muscle due to painful stimulation may result in an ongoing change in upper limb motor control. As sensorimotor disturbances are known to persist beyond the acute episode of pain [44] and are thought to play a defining role in the clinical picture and chronicity of different chronic neck pain conditions [45], then the changes in central activation without changes in MVC observed in the current study after spinal manipulation may reflect a normalization of such injury-/pain-induced central plastic changes, which may reflect one mechanism for the improvement of functional ability reported after spinal manipulation. This is supported by previous studies demonstrating that spinal manipulation can alter sensorimotor integration at the cortical level [13], most likely occurring within the pre-frontal cortex [37], and/or change the communication between the cerebellum and the primary motor cortex (M1) [19,20], all of which could account for the current study findings of altered cortical drive originating from pre-M1 [13,37]. Multiple studies have demonstrated changes in somatosensory evoked electroencephalographic potentials (SEPs) following manipulation of dysfunctional spinal joints [13,46], the most recent of which also used a source localization method and demonstrated that these changes in the N30 SEP peak occur within the prefrontal cortex [37]. Also, VA measured with TMS showed a significant increase after spinal manipulation, whereas no changes were observed in VA measured using peripheral nerve stimulation. In light of this finding, it is possible that the increase in voluntary activation of the elbow flexors observed in this study following spinal manipulation occurred due to changes at the cortical level possibly at prefrontal cortex site as its highly involved motor control [47], and is altered by spinal manipulation of dysfunctional joints [37].

The population for this study was very carefully chosen. Multiple studies have shown that people with a previous history of recurring ache, pain or tension in the neck, even though they were pain-free on the day of the experiment, display reduced proprioceptive awareness of their elbow joint [15] and shoulders [48]. Subclinical neck pain people also display more head trunk and whole body movements when their shoulders are lifted [48]. Their cervical spines fatigue faster, and they have lower cervical flexion relaxation ratios [49]. This suggests that episodes of spinal problems, if not treated in any way (i.e., that is the definition of subclinical pain populations, that they have a history of recurrent neck ache pain or tension that they have not yet sought treatment for and that they are pain free on the day of the experiment) may potentially influence spinal and body proprioceptive awareness and motor control. This is likely due to altered afferent information from the affected areas occurring beyond the episodes of symptoms that leads to maladaptive plastic changes within the central nervous system. Numerous animal studies have shown that experimentally injuring the spine will, over time, lead to specific changes in the small paraspinal muscles surrounding the injured area [50,51,52,53]. For example, multifidus muscle fibrosis, fatty infiltration and changes in muscle fibre types from slow-to-fast twitch types have been shown to occur within weeks to months post-injury in experimental animal models [50,51,52,53]. Human studies have also shown fatty infiltration in multifidus muscles does occur over time in those who have had episodes of back pain [54,55,56,57], and multifidus muscle atrophy has been found in individuals with chronic spinal degeneration [58]. This body of research has led some authors to conclude that spinal pain-induced disrupted or reduced proprioceptive signalling likely plays a pivotal role in driving long-term changes in the central nervous system leading to motor and cortical sensory reorganization, and that these spinal proprioceptive changes are vital in the recurrence and chronicity of spinal pain conditions [59]. One likely location within the CNS where such plastic changes may be occurring is within the cerebellum. It is well known that the cerebellum receives and integrates large amounts of sensory information from joints, tendons and muscles, including those from the intervertebral regions of the neck [60] as well as nociceptive inputs [61]. Alterations in proprioceptive input from the neck over a longer period from episodes of spinal dysfunction or pain may alter the way the cerebellum integrates other sensory information and alter motor control of these same structures. Support for this theory comes from TMS and SEPs studies that have shown that subclinical neck pain people display differences in cerebellums-M1 communication [19] as well as cerebellum-S1 communication [62], respectively, when compared to individuals that have never had any spinal complaints. This distorted sensorimotor integration appears to respond well to spinal manipulation. However, the body of research exploring this is still limited.

Spinal manipulation has also been shown to decrease the TMS-induced cortical silent period of motor neurons to the upper limb [40,63]. Also, using TMS induced stimulus responses curves, spinal manipulation has been shown to increase the maximum TMS-induced motor evoked potentials (MEPs) of both upper and lower limb muscles [64]. In this same study, in an effort to identify where the changes in MEP amplitudes were coming from, movement related cortical potentials (MRCPs) were also recorded. They showed that after spinal manipulation, the early part of the MRCP increased significantly [64]. This early part of the MRCP is known to be generated by cortical and subcortical structures [65]. Spinal manipulation has also been shown to increase intra-cortical facilitation and decrease intra-cortical inhibition [40]. Additionally, people with subclinical neck pain have been shown to have altered cerebellar-motor cortex inhibition that can be improved with spinal manipulation [19,20]. Taken together, there is a growing body of evidence that suggests that spinal manipulation of dysfunctional joints can alter sensorimotor integration in a manner that improves motor output. In addition, these changes appear to reside at or above the level of the motor cortex.

### Limitations and Future Directions

It is important to note that throughout the experiment, the investigators continuously monitored the amplitudes of MEPs in real-time to ensure large biceps MEPs were consistently elicited with minimal triceps MEPs in all subjects, in all sessions. However, it would have been useful to have recorded and saved this data for offline analysis. Unfortunately, this was not done for this study. The functional relevance of a small increase in voluntary activation after spinal manipulation is currently unclear, and the time course of this change remains to be elucidated. It is noteworthy that only one of our study participants was left-handed (1/18), as such, we cannot conclusively eliminate potential influence of hand dominance in our results. However, it is unlikely that hand dominance would dramatically influence the neurological effects of spinal manipulation. It is also unclear whether the increase in motor cortical drive after spinal manipulation can be generalized to other muscle groups. It is also possible that the increase in voluntary activation could have been underestimated because the elbow flexor muscles are relatively well-activated. That is, the increase in voluntary activation may be more profound in less-activated muscles (such as the abductor digiti minimi and brachioradialis). Also, results regarding origin of changes should be interpreted with caution as peripheral nerve stimulation protocol was not completed by all the participants. The cumulative effects of spinal manipulation on voluntary activation also warrant further investigation, and it would be good to explore whether peripheral joint manipulation has a similar effect to spinal manipulation. Lastly, it would be of interest to follow-up this study in a population that has decreased cortical muscle activation efficiency, such as patients who have lost tonus of their muscles and/or are recovering from muscle degrading dysfunctions such as stroke or orthopedic operations.

## 5. Conclusions

A single session of spinal manipulation in participants with subclinical neck pain resulted in an immediate increase in voluntary activation of the elbow flexors. The site of neural adaptation post-manipulation must have occurred at or above the level of the motor cortex.

## Figures and Tables

**Table 1:** Baseline and post mean values.

(Spinal Manipulation)	(Passive Movements)	(Spinal Manipulation)	(Passive Movements)	(Spinal Manipulation)	(Passive Movements)
MVC (Kg’s)	MVC (Kg’s)	VA Cortical (TMS)	VA Cortical (TMS)	VA Peripheral Nerve Stimulation)	VA Peripheral Nerve Stimulation)
Pre 20.37 (SD = 7.36)	20.14 (SD 7.39)	88.80 (SD 8.17)	91.36 (SD 7.39)	89.5 (SD 13.22)	94.74 (SD 5.68)
Post 20.84 (SD = 7.345)	19.89 (SD 7.41)	91.47 (SD 7.16)	89.70 (SD 12.26)	90.77 (SD 9.28)	93.83 (SD 7.50)
